# Invasive Group A Streptococcal Infections: Benefit of Clindamycin, Intravenous Immunoglobulins and Secondary Prophylaxis

**DOI:** 10.3389/fped.2021.697938

**Published:** 2021-08-20

**Authors:** Delphine Laho, Sophie Blumental, Anne Botteaux, Pierre R. Smeesters

**Affiliations:** ^1^Paediatric Department, Academic Children Hospital Queen Fabiola, Université Libre de Bruxelles, Brussels, Belgium; ^2^Molecular Bacteriology Laboratory, Université Libre de Bruxelles, Brussels, Belgium; ^3^Tropical Diseases Research Group, Murdoch Children's Research Institute, Melbourne, VIC, Australia; ^4^Department of Paediatrics, University of Melbourne, Melbourne, VIC, Australia

**Keywords:** invasive group A streptococcal infections, clindamycin, immunoglobulins, secondary prophylaxis, chemoprophylaxis

## Abstract

**Introduction:** Mortality associated with invasive group A streptococcal infections (iGAS) remains high among adults, with lower mortality in children. The added value of both clindamycin and immunoglobulins in such treatment is still controversial, as is the need for antibiotic secondary prophylaxis. It is unlikely that conclusive randomized clinical studies will ever definitively end these controversies.

**Materials and Methods:** A clinical and experimental literature review was conducted in Pubmed, Cochrane, and lay literature to determine the benefit of adding clindamycin and immunoglobulins to β-lactams in the management of iGAS, as well as the need for secondary prophylaxis measures in close contacts.

**Results:** This review includes two meta-analyses, two randomized controlled trials, four prospective studies, five retrospective studies, and microbiological studies. To reduce mortality and morbidity, it appears useful to add clindamycin to β-lactams in severe clinical presentations, including necrotizing fasciitis or streptococcal toxic shock syndrome, and immunoglobulins for the latter two presentations. The high risk of secondary infection in household contacts justifies the need of taking preventive measures.

**Conclusions:** Both clinical studies and available experimental evidence suggest that adding clindamycin and immunoglobulins as adjunctive therapies in the management of invasive group A streptococcal infections may reduce mortality. Household contacts should be warned about the increased risk of secondary infection, and chemoprophylaxis may be considered in certain situations.

## Introduction

Group A Streptococcus (GAS) causes a wide spectrum of clinical syndromes ranging from asymptomatic carriage to life-threatening infections. Invasive Group A Streptococcal infections (iGAS) are defined by the isolation of GAS from a normally sterile site (e.g., blood, pleural, or cerebrospinal fluid) with or without clinical evidence of invasive diseases or a deep-seated infection [necrotising fasciitis (NF), pneumonia, osteomyelitis] (1). IGAS affect 663,000 people each year globally and cause 163,000 deaths (2). Although GAS is always sensitive to penicillin [even if some mutations in penicillin-binding protein genes conferring reduced susceptibility to β-lactam antibiotics have been reported (3)], the mortality from these infections remains high, especially among adults which can reach up to 24% for NF and 36% for streptococcal toxic shock syndrome (STSS) (4), with lower mortality in children in high-resource settings. Numerous virulence factors have been shown to contribute to GAS virulence. Exotoxins that act as superantigens and activate the immune system were associated with several clinical syndrome including STSS (1, 5). The M protein promotes GAS infection by various means including the inhibition of phagocytosis (6). GAS also produces enzymes that prevent GAS from being killed like SpeB, a protease degrading host and bacterial components (7), Sda1, a DNAse destroying neutrophils extracellular traps (8), and toxins like Streptolysin O (SLO) cytotoxic for macrophages and neutrophils (6). Appropriate diagnostic and rapid treatment based on β-lactam antibiotics and supportive care are the most important factors in reducing mortality (9). However, variety of treatment protocols exists particularly in the potential use of adjuvant therapies such as clindamycin and intravenous immunoglobulins (IVIG) (Table 1). Although their use is overall supported by both biological and microbiological experimental data, as well as by observational studies, conclusive clinical data supporting their efficacy in reducing iGAS mortality remain limited. In addition, an increased risk of secondary iGAS infections in close contacts from the index case has been described (10, 19). Whether antibiotic secondary prophylaxis allows for a reduction in secondary cases remains uncertain. We aimed to summarize the available experimental and clinical evidence about the efficacy of adding clindamycin and IVIG to β-lactams in the management of iGAS. We also aimed to assess the benefits and optimal regimen of antibiotic prophylaxis in close contacts of patients.

**Table 1:** Guideline.

**Countries/Recommendations**	**IVIG**	**Clindamycin**	**Secondary prophylaxis**
Common recommendations	None	None	• For all: inform close contacts • Seek medical attention promptly if symptoms occur • Antibiotics to close contacts if they present any symptoms of a localized infection with GAS (angina, fever, skin infection, etc.) (1, 10–13)
USA	Infection refractory to aggressive treatment or a non-drainable focus or an oliguria persistence with pulmonary oedema (14)	Severe GAS infection^◦^ (14)	• Chemoprophylaxis to household contacts who have a high risk of iGAS (age ≥ 65 years, HIV infection, diabetes, cancer, heart disease, addiction, corticosteroids, Native American origin), or death (10, 14) • Penicillin + rifampicin • Clindamycin • Azithromycin (10, 14)
Canada	Severe GAS infection^◦^ or infection refractory to aggressive treatment (15)	Severe GAS infectio^◦^ (15)	• Chemoprophylaxis to close contacts of a patient with a severe iGAS^◦^ • Chemoprophylaxis to close contacts if two or more cases occur in a community within 1 month • chemoprophylaxis to close contacts if a case in a child care center occurs at the same time as a chickenpox outbreak • 1st choice: 1st generation cephalosporins • If beta-lactams allergy: clindamycin or macrolides (15)
United Kingdom	No consensus	No consensus	• Chemoprophylaxis to a mother or her child if either has an iGAS during the neonatal period (the first 28 days of life) • Chemoprophylaxis to close contacts if two or more cases occur in a community within 1 month • 1st choice: oral penicillin • If beta-lactams allergy: azithromycin (12)
Ireland	STSS or NF if associated with organ failure (16)	Suspected severe infection^◦^ (16)	• Chemoprophylaxis to a mother or her child if either has an iGAS during the neonatal period (the first 28 days of life) • Chemoprophylaxis to close contacts if two or more cases occur in a community within 1 month • 1st choice: oral penicillin • If beta-lactams allergy: azithromycin (16)
France	STSS or NF	NF, STSS, or toxin signs (rash, digestive or hemodynamic disorders) (17)	• Chemoprophylaxis to close contacts at risk of iGAS or complications (age ≥ 65 years, chickenpox, extensive skin lesions (including burns), drug addiction, progressive pathology (diabetes, cancer, hematology, HIV infection, heart failure), oral corticosteroid treatment (defined as doses > 5 mg/kg/day prednisone for more than 5 days or doses equivalent to or >0.5 mg/kg/day prednisone for ≥30 days) • 1st choice: 2nd or 3rd generation cephalosporins • If beta-lactams allergy: clindamycin or macrolides • If macrolide-resistant GAS: oral penicillin + rifampin (13)
Australia	No consensus	No consensus	• Chemoprophylaxis to close contacts of patient with a severe iGAS^◦^ • Chemoprophylaxis to a mother or her child if either develops an iGAS in the neonatal period (the first 28 days of life) • Chemoprophylaxis to close contacts if two or more cases occur in a community within 3 months • 1st choice: benzathine penicillin (intramuscular) • 2nd choice if oral therapy preferred: cephalexin • If beta-lactams allergy: macrolides • If macrolide-resistant GAS or pregnant women: clindamycin (11)
Belgium (Flanders)	No consensus	No consensus	• Chemoprophylaxis to all household contacts of the index case • 1st choice: azithromycin • If macrolide-resistant GAS or pregnant women: clindamycin (18)
Our recommendations	All hemodynamically unstable patients and/or admitted to intensive care unit and/or having STSS or NF	For all hospitalized iGAS infections	• Chemoprophylaxis to all household members of the patient • Chemoprophylaxis to people at high risk of complications or deaths related to iGAS • 1st choice: first-generation cephalosporins • If beta-lactams allergy: macrolides • If macrolide-resistant GAS or pregnant women: clindamycin

◦ *Severe iGAS refers to iGAS such as pneumonia, meningitis, NF, STSS, or any manifestation requiring admission to intensive care or leading to death*.

## Materials and Methods

A clinical and experimental literature review was conducted in Pubmed, Cochrane, and lay literature (governmental websites, pharmacology website, and books) using the following keywords: ≪ (group A streptococcus OR Streptococcus pyogenes) AND (invasive infections OR toxic shock syndrome OR necrotizing fasciitis) AND (treatment OR immunoglobulin OR clindamycin OR secondary OR prophylaxis OR prophylactic OR contact OR family OR household).≫ The articles were selected regarding their language (English and French) without any time limit. The initial search resulted in 1,438 articles; among which, 1,293 were excluded based on their title and abstract (irrelevant articles about other treatments than clindamycin and IVIG, other pathogens than GAS, non-invasive streptococcal diseases, and low-quality studies). We also checked the references of the selected articles for relevant articles. In total, 149 articles were read entirely and 59 articles were included in this review.

### Benefit of Intravenous Immunoglobulins in iGAS Management

The rationale for adding intravenous immunoglobulins (IVIG) to iGAS treatment is based on the major inflammatory response leading to systemic toxicity and multiple organ failure. The trigger for this excessive reaction relies on both host factors (genetic influence, absence of prior immunity against GAS) and the characteristics of the pathogen [expression of certain M proteins, DNases (20), and superantigens]. IVIG consist of a mixture of human IgG antibodies collected from thousands of donors. In adjuvant therapy, IVIG could act by promoting opsonization and phagocytosis of bacteria, neutralizing toxins, and exerting an immunomodulatory effect (21).

In addition, the actions of IVIG have been demonstrated *in vitro* and in mice experimental models, including neutralization of circulating superantigens and reduction of the systemic inflammatory response (22, 23). A recent study analyzed the effects of IVIG on virulence factor activity in three different ways: *in vitro, in vivo* in a murine infection model, and *ex vivo* in patients (24). *In vitro*, SLO and Sda1 activities were reduced. In the murine infection model, mice treated with IVIG had smaller skin lesions and a lower SLO activity. Moreover, serum from patients with iGAS had a reduced SLO and Sda activity after IVIG was administrated compared with before such treatment (24). In addition, plasma of STSS-patients treated with IVIG was able to neutralize streptococcal superantigens and completely inhibit cytokine production (25), suggesting a clinical interest of such adjunctive therapy for these patients. A recent study demonstrated that one 25-g IVIG dose was sufficient to achieve plasma neutralization of GAS superantigenic activity. The study showed a negative correlation between IVIG dose and toxin-triggered T-cell proliferation (*r* = −0.67, *p* < 0.0001) (26). It also demonstrated a strain-dependant variation in the IVIG effect (26).

As shown in Table 2, several studies have been conducted to evaluate the benefit of IVIG in iGAS management. Two prospective studies of 53 Canadian and 67 Swedish STSS patients reported that IVIG use was associated with a lower mortality [respectively, survival OR: 8.1 (95% IC, 1.6–45; *p* = 0.009) (27) and OR survival: 5.6; IC 95%: 1.2–2.9, *p* = 0.03 (21)]. In addition, a multicenter, randomized, placebo-controlled trial in 17 hospitals on 21 patients with STSS, with or without necrotizing fasciitis, evaluating the efficacity of adding IVIG to the bitherapy penicillin-clindamycin demonstrated a higher mortality in the group who did not receive IVIG (Death rate: 3.6 times higher in the placebo group, *p* = 0.3) (28). This finding was statistically non-significant, probably because of the small number of patients included. Initially, the study was designed to include 120 patients but the slow patient recruitment interrupted the trial prematurely (28). A retrospective study on 322 patients with NF failed to show any benefit of IVIG regarding mortality or hospital length of stay (29). Nevertheless, the patients who received IVIG were much more ill, which could have underestimated a positive effect of IVIG (29). Recently, a blinded, randomized, placebo-controlled clinical trial assessing the effect of IVIG vs. placebo in 100 adult patients with necrotizing soft tissue infections did not demonstrate any effect on self-reported physical functioning at 6 months nor in mortality or organ failure [Risk Ratio (RR) mortality: 0.80; IC 95% 0.40–1.59; *p* = 0.65] (30). However, the potential effect of IVIG could have been hidden by the single dose of IVIG received by nearly half of the patients in the placebo group before the randomization (30). A single dose of 25-g IVIG indeed has an effect on GAS superantigenic activity (26).

**Table 2:** Studies assessing the clinical efficacy of IVIG and clindamycin in iGAS.

**References**	**Design study**	**Number of patients**	**Type of patients**	**Treatments compared**	**Results**	**Conclusions**
**IVIG**						
Carapetis et al. (19)	Prospective- retrospective	84	Children and adults (Average age of the two groups compared: 56.2 vs. 70.4 years)	Clindamycin vs. no clindamycin; analyzed subgroup: clindamycin + IVIG vs. clindamycin without IVIG	OR mortality: 0.12 (95% CI: 0.1–1.29^#^)	IVIG further reduces mortality when added to clindamycin in case of STSS or NF
Linnér et al. (21)	Prospective	67	Adults (Average age of the two groups compared: 60 vs. 65 years)	IVIG vs. no IVIG	OR survival: 5.6 (95% CI: 1.2–2.9, *p* = 0.03^*^)	IVIG decrease mortality in STSS
Kaul et al. (27)	Prospective	53	Adults (Average age of the two groups compared: 52 vs. 60 years)	IVIG vs. no IVIG	OR survival: 8.1 (95% CI: 1.6–45, *p* = 0.009^*^)	IVIG decrease mortality in STSS
Darenberg et al. (28)	A multicentre, randomized, double-blind, placebo-controlled	21^a^	Adults (Average age of the two groups compared: 51.3 vs. 52.6 years)	IVIG vs. placebo	Death rate: 3.6 times higher in the placebo group (*p* = 0.3^#^)	IVIG seem to decrease mortality in STSS
Kadri et al. (29)	Retrospective	322	Adults (Average age of the two groups compared: 48.8 vs. 54 years)	IVIG vs. no IVIG	Same mortality (*p* = 0.99^#^) and hospital length of stay (*p* = 0.84^#^) in the two groups	No difference on mortality or hospital length of stay in NF
Madsen et al. (30)	Randomized, blinded, placebo-controlled clinical trial (INSTINCT)	100	Adults (Average age of the two groups compared: 59 vs. 61 years)	IVIG vs. placebo	No effect on self-reported physical functioning at 6 months (*p* = 0.81^#^) Same mortality in the two groups	No difference on physical functioning or mortality in NF
(31)	Retrospective	192	Children (Average age: 8.8 years)	IVIG vs. no IVIG	Same mortality in the two groups (p = 1^#^)	No difference in mortality in STSS
Adalat et al. (32)	Prospective (survey which results are based on participating centers only)	49	Children (Average age: 4.8 years)	IVIG vs. no IVIG	No death in the IVIG group	IVIG decrease mortality in STSS
Alejandria et al. (33)	Meta-analysis	1958	Children and adults (Average age not mentioned)	IVIG vs. placebo or no intervention	RR mortality: 0.77 (95% CI: 0.68–0.87^*^)	IVIG decrease mortality in adults with septic shock, no matter the bacteria involved
Parks et al. (34)	Meta-analysis	165	Children and adults (Average age not mentioned)	IVIG vs. no IVIG in clindamycin-treated patients with STSS	RR mortality: 0.46 (95% CI: 0.26–0.83^*^)	IVIG decrease mortality in STSS
**Clindamycin**						
Carapetis et al. (19)	Prospective	84	Children and adults (Average age of the two groups compared: 56.2 and 70.4 years)	β-lactam + Clindamycin vs. onlyβ-lactam	OR mortality: 0.31 (95% CI: 0.09–1.12^#^)	Combination of β-lactam + clindamycin decreases mortality in case of STSS or NF
Zimbelman et al. (35)	Retrospective	56	Children (Average age: 3.8 years)	Clindamycin (±β-lactam) vs only β-lactam	Favorable outcome: 83% (vs. 14%) (*p* = 0.006^*^)	Patients with deep infection (NF, bacteremia, arthritis, osteomyelitis) were more likely to have a favorable outcome if initial treatment included clindamycin
Mulla et al. (36)	Retrospective	195	Adults (Average age: 52 years)	Only clindamycin or in combination Vs. no clindamycin	OR mortality in NF: 0.11 (95% CI: 0.01–0.89^*^)	Clindamycin decreases mortality in case of NF
Linnér et al. (21)	Prospective	67	Adults (Average age of the two groups: 60 vs. 65 years old)	Clindamycin vs. no clindamycin	OR survival: 8.6 (95% CI: 1.8–40.4, *p* = 0.007^*^)	Clindamycin decrease mortality in STSS
Babiker et al. (37)	Retrospective	1,956	Adults (Average age of the two groups compared: 48 vs. 47 years)	Clindamycin vs. no clindamycin	OR mortality: 0.44 (95% IC: 0.23–0.81^*^)	Clindamycin decrease mortality in case of iGAS with or without shock or NF

*OR stands for odds ratio; RR stands for relative risk; 95% CI stands for 95% confidence interval*;

* *stands for significant result*;

# *stands for non-significant result*

a *120 patients were initially planned but the trial was prematurely terminated due to the low incidence of STSS in the participating countries and the slow patient recruitment. As a result, only 21 patients were included*.

Focusing on a pediatric setting, only a few studies were published in children with iGAS. Children with STSS had a lower mortality rate than adults making demonstration of a benefit in clinical studies even more complicated (4). An American retrospective study on 192 children with STSS did not show any difference in mortality whether IVIG was used or not (4.5% in both groups, *p* = 1) (31). However, the low mortality rate observed in both groups interrogates about the STSS case definition and severity of included patients. In a multicenter, retrospective 2014 study on 49 children, IVIG was not given to children who died and no death happened in the IVIG-group (32). However, as this study was based on a questionnaire sent to institutions that first agreed to participate and not all reported back, there may have been a higher risk of methodological bias (32). An Australian prospective study on children and adults found a decrease in mortality when IVIG were added to penicillin and clindamycin (19). A Cochrane review published in 2013 on adults and children, including 17 randomized controlled trials, showed the use of IVIG decreases the mortality in cases of sepsis in adults (RR 0.77; 95% CI 0.68–0.87), irrespective of the pathogen responsible which limits the impact on GAS specific sepsis (33). A subanalysis including only studies considered to have a lower risk of bias did not retrieve the same conclusion in adults and neonates (RR 0.97; 95% CI 0.81–1.15; *n* = 945) (33). A meta-analysis conducted by Parks et al., including studies on adults and children, showed that the IVIG on clindamycin-treated patients significantly reduced STSS related-mortality from 33.7 to 15.7% (RR: 0.46; IC 95%: 0.26–0.83; *p* = 0.01) (34). Nevertheless, some biases are possible due to non-RCT studies included in this meta-analysis (34).

Current recommendations regarding IVIG treatment in iGAS vary (Table 1). The American Academy of Pediatrics recommend the use of IVIG in case of infection refractory to aggressive treatment or a non-drainable focus or an oliguria persistence with pulmonary edema (14), whereas the French Society of Pediatrics advises the use of IVIG in case of STSS or NF (17). In Ireland, IVIG are considered for STSS or NF if associated with organ failure (16). The dosages used are 1 g/kg on the first day then 0.5 g/kg for the following 2 days. However, no study has been performed to define the optimal dosage or schedule of administration (14). Side effects are rare, with anaphylactic reaction being the most serious (38). Moreover, IVIG cost is not negligible (45 euros per gram) (38). However, regarding their efficacy and the low risk of side effects, we recommend the administration of IVIG in all hemodynamically unstable patients and/or admitted to intensive care unit and/or having STSS or NF. Hemodynamic status has to be evaluated before IVIG administration as it can be compromised in critically ill patients who may not tolerate large amount of liquids.

### Benefit of Clindamycin in iGAS Management

The adjunction of a second antibiotic, clindamycin, to the conventional beta-lactam therapy is widely used and has been evaluated by several *in vitro* and *in vivo* studies (35). Clindamycin has an excellent tissue penetration *in vitro* and a long effect after administration. Moreover, it remains active regardless of the size of the bacterial inoculum or growth stage (3). This contrasts with β-lactams antibiotics which are known to be less efficient when the bacterial inoculum increases so much that most of them are in stationary replication phase (Eagle effect) (39). Clindamycin therefore acts synergistically with β-lactams to reduce the bacterial load in iGAS (40).

Clindamycin inhibits protein synthesis by binding to the 50S subunit of the bacterial ribosome. Clindamycin therefore blocks the transcription and production of many virulence factors involved in systemic toxicity and tissue destruction such as the M proteins, superantigens, streptolysins, and DNases (41). In mice experiments comparing clindamycin vs. placebo, clindamycin has been shown to decrease the expression of DNase (Sda1) and SLO that was associated with a reduction of the cutaneous lesions size (42). In another recent experimental paper using an iGAS mice model, clindamycin improved the survival rate of infected mice and the frequency of immune cells involved in host infection defense (7).

Furthermore, some clinical studies evaluated the impact of adding clindamycin in the management of iGAS in humans (Table 2). Two studies, one including 195 adult patients with NF and another 62 adult patients with a STT, found a significant decrease in mortality in clindamycin-treated patients [respectively, odds ratio (OR) mortality: 0.11; 95% IC: 0.01–0.89 (36); OR survival: 8.6; 95% IC: 1.8–40.4 *p* = 0.007] (21). Another recent paper about 1,956 adults showed a significantly lower mortality in patients with iGAS who received clindamycin even if they did not have a vasopressor-dependent shock, NF, or both (OR mortality: 0.44; 95% IC: 0.23–0.81) (37).

Two studies included pediatric patients. A retrospective study of 56 children in the USA showed a statistically significant decrease in mortality when clindamycin was added to a β-lactam for GAS deep-sited infection (survival with clindamycin: 83 vs. 14% without clindamycin, *p* = 0.006) (35). An Australian prospective study following 4.9 million people over the 2.5-year reported 84 iGAS cases (age: 3.8–88.1 years) and suggested the effectiveness of adding clindamycin on mortality (OR mortality: 0.31; IC 95%:0.09–1.12) (19). However, their results did not reach a statistical significance, probably because of the small number of patients included in the study (19). Despite the convincing results of these observational surveys, the lack of conclusive randomized trials led to a great variability in expert recommendations. The American Academy of Pediatrics and the Canadian Pediatric Society recommend adding clindamycin to β-lactams (30–40 mg/kg/day in 3–4 doses intravenous, maximum 1.8–2.7 g/day) in case of a severe GAS infection such as pneumonia, meningitis, NF, STSS, or any manifestation requiring admission to intensive care or leading to death (14, 15). In Ireland, clindamycin is prescribed as soon as a severe infection is suspected (16). The French Society of Pediatrics suggests the use of clindamycin in case of NF, STSS, or toxin signs (rash, digestive, or hemodynamic disorders) (17). However, indications in other severe cases remain a matter of debate. As clindamycin is a relatively safe and inexpensive molecule (38), we propose to use a β-lactam-clindamycin combination to treat all serious iGAS infections which need to be treated in a hospital.

### Benefit of Secondary Antibiotics Prophylaxis

Outbreaks of iGAS have been reported in homecare facilities, hospitals, schools, as well as in close contacts of a patient who presented a recent iGAS episode (15, 43, 44). Adults experiencing homelessness are also at risk of iGAS (44, 45). These people cumulate risk factors for GAS colonization, such as crowding and skin breakdown and often underlying medical conditions and alcohol abuse (44–46). Definition of close contacts for iGAS includes people who were closely in contact with the index case during the seven days preceding the onset of symptoms and up to 24 h after the onset of antibiotic therapy in the index case. The close contacts shared the same home; had intimate physical contact (people who have had sex or spent at least 50% of the nights in the same house); persons attending institutions (child-care, homes for the elderly, prisons, military camps, etc.) and staff members; persons who have had direct physical contact via mucous membrane or damaged skin with nasal or pharyngeal secretion of the index case (10, 11, 15, 46).

Five population-based studies evaluated the risk of secondary infections in close contacts of a patient who developed an iGAS and all found an increased risk compared to the general population. Four studies performed in England, USA, Canada, and Australia demonstrated an increased risk ranging from 229 to 2,011 times higher for household contacts compared to the general population (19, 47–49). Another UK study published in 2017 found an increased odds to develop an iGAS for a mother or her newborn if either of them has an iGAS during the first 28 days after delivery with a 1,940 (95% CI: 1,240–2,880)-fold increase (50).

The risk of secondary iGAS in close contacts is higher within the first 7 days and remains high until 30 days after the last contact with the index case (19, 43, 47, 49). All the papers above unanimously demonstrated that close contacts of an iGAS patient are more prone to develop the infection compare to the general population, similarly to what is observed in the case of meningococcemia (51). However, in contrary to meningococcal diseases for which the prescription of antibiotics prophylaxis to close relatives is commonly admitted, there is no consensus regarding secondary prophylaxis after iGAS (52). Even though the risk of iGAS among close contacts is higher than the general population, it remains low (12). In addition, no study has demonstrated a reduction in the risk of iGAS in close contacts after a chemoprophylaxis. The effective treatment of pharyngitis or oropharyngeal carriage of GAS has been proven with 10 days of penicillin associated with rifampin (4 days) or 10 days of clindamycin (53, 54). Cephalosporins have also been shown to be more effective than penicillin for pharyngitis in GAS (55, 56). In addition, an important fear is that taking chemoprophylaxis may falsely reassure patients and delay their presentation to the emergency room (57). It would be useful to carry out studies on the effect of chemoprophylaxis administered to at-risk groups on the occurrence of secondary cases of iGAS and to monitor whether these attitudes will lead to the emergence of resistance.

The Centers for Disease Control and Prevention (CDC), the Superior Council of Public Hygiene of France, the British Infectious Disease Association, and the Center for Disease Control in the Northern Territory of Australia recommend to inform close contacts about the potential risk of developing iGAS, notably by explaining the clinical manifestations of pharyngeal or invasive GAS infections and the importance of prompt medical consultation in case these symptoms will appear during the month following infection in the index case (10–13). It is also advisable to treat close cases if any of them has a localized infection such as pharyngitis, skin infection, and/or fever (1, 12). Performing a throat swab to assess GAS carriage is considered useless to guide chemoprophylaxis (15). In some cases, a chemoprophylaxis can be proposed (Table 1). Indications for antibioprophylaxis widely differ according to the regions of the world but everyone agrees that if a chemoprophylaxis is prescribed, it should be administered as quickly as possible, ideally within the 24 hours after the diagnosis of the index case and up to 7 (15) to 30 days (11) after the last contact. The GAS colonization increase in close contacts who were exposed at least 24 h/week to the index patient (36 vs. 2%) (58).

In the USA and France, a chemoprophylaxis is recommended only for household contacts with a high risk of iGAS (age ≥ 65 years, HIV infection, diabetes, cancer, heart disease, addiction, corticosteroids, Native American origin) or death (10, 13, 14). In Canada, a chemoprophylaxis is prescribed for all close contacts of patients with a severe iGAS (regardless of his underlying medical conditions) or to the members of a community where two or more cases occurred within the same month (15). In Great Britain and Ireland, a chemoprophylaxis is given to a mother or her child if either has presented with suspected or confirmed iGAS during the neonatal period (the first 28 days of life) or to all members of a community where two or more cases occurred within the same month (12, 16). The theoretical number needed to treat (NNT) to prevent one secondary case using antibiotic prophylaxis was evaluated to 271 overall (95% CI: 194–454) and to 50 for mother-neonate pairs (95% CI: 27–393) (50). In Australia, a chemoprophylaxis will be advised in three kinds of situations: for all close contacts of a patient with a severe iGAS, for a mother or her child if either has a suspected or confirmed iGAS within 28 days of birth, and for all asymptomatic institutional contacts if two or more cases occurred in a community within 3 months (11). Finally, in Belgium and particularly in Flanders, a chemoprophylaxis is prescribed to all household members of the index case (18). We recommend that all contacts of an iGAS patient must be informed about the higher risk of developing iGAS and the importance of prompt medical consultation if symptoms appear during the following month. For serious iGAS requiring treatment at a hospital, we also recommend chemoprophylaxis for all household members of the patient including mother-baby couples who have the highest risk of secondary iGAS. We also recommend prophylaxis for people at a high risk of complications or deaths related to iGAS, both children and elderly people (Table 1). We suggest using a first-generation cephalosporins for 10 days [cefadroxil 30 mg/kg/day (maximum daily dose, 2 g/day) in two or three divided doses] and keep azithromycin for 3 days [10 mg/kg/day (maximum daily dose, 500 mg/day) in a single dose] only for people who are allergic to beta-lactams (59). We do not recommend testing for macrolide sensitivity to avoid chemoprophylaxis administration delay and because of the relatively low frequency of macrolides resistance in most settings. Nevertheless, if the GAS strain isolated from the index case is found to be resistant to macrolides or for pregnant women, clindamycin should be prescribed for 10 days [20 mg/kg/day (maximum daily dose, 900 mg/day) in three divided doses]. Regarding caregivers, as the contact with the patient occurs <24 h per week, the risk of secondary iGAS is probably low (58).

## Conclusions

Conclusive clinical evidence regarding the benefit of adjunctive clindamycin, IVIG, and secondary prophylaxis for iGAS patient are unlikely to arise. However, a convergent body of *in vitro, in vivo*, and *ex vivo* animal and human clinical data suggest that clindamycin and IVIG should be used as adjunctive therapies when possible. Physicians must also be warned of the increased risk of iGAS in close contacts for 28 days and should inform the close contacts of their patients. Antibiotic prophylaxis in such iGAS close contacts may reduce the risk of developing the disease.

## Author Contributions

PS and DL contributed to conception of the review. DL wrote the first draft of the manuscript. PS made important improvements of the manuscript. All authors contributed to manuscript revision, read, and approved the submitted version.

## Conflict of Interest

The authors declare that the research was conducted in the absence of any commercial or financial relationships that could be construed as a potential conflict of interest.

## Publisher's Note

All claims expressed in this article are solely those of the authors and do not necessarily represent those of their affiliated organizations, or those of the publisher, the editors and the reviewers. Any product that may be evaluated in this article, or claim that may be made by its manufacturer, is not guaranteed or endorsed by the publisher.
